# Metabotropic Glutamate Receptors for Parkinson's Disease Therapy

**DOI:** 10.1155/2013/196028

**Published:** 2013-06-19

**Authors:** Fabrizio Gasparini, Thérèse Di Paolo, Baltazar Gomez-Mancilla

**Affiliations:** ^1^Novartis Pharma AG, Novartis Institutes for BioMedical Research Basel, Forum 1, Novartis Campus, 4056 Basel, Switzerland; ^2^Neuroscience Research Unit, Centre Hospitalier Universitaire de Québec, CHUL, Quebec City, QC, Canada G1V 4G2; ^3^Faculty of Pharmacy, Laval University, Quebec City, QC, Canada G1K 7P4

## Abstract

Excessive glutamatergic signalling within the basal ganglia is implicated in the progression of Parkinson's disease (PD) and inthe emergence of dyskinesia associated with long-term treatment with L-DOPA. There is considerable research focus on the discovery and development of compounds that modulate glutamatergic signalling via glutamate receptors, as treatments for PD and L-DOPA-induced dyskinesia (LID). Although initial preclinical studies with ionotropic glutamate receptor antagonists showed antiparkinsonian and antidyskinetic activity, their clinical use was limited due to psychiatric adverse effects, with the exception of amantadine, a weak N-methyl-d-aspartate (NMDA) antagonist, currently used to reduce dyskinesia in PD patients. Metabotropic receptor (mGlu receptor) modulators were considered to have a more favourable side-effect profile, and several agents have been studied in preclinical models of PD. The most promising results have been seen clinically with selective antagonists of mGlu5 receptor and preclinically with selective positive allosteric modulators of mGlu4 receptor. The growing understanding of glutamate receptor crosstalk also raises the possibility of more precise modulation of glutamatergic transmission, which may lead to the development of more effective agents for PD.

## 1. Introduction

Parkinson's disease (PD) is a chronic progressive neurodegenerative disorder of the central nervous system (CNS), characterised by a gradual loss of dopaminergic neurotransmission. Cardinal symptoms of PD include tremor, bradykinesia, and rigidity. Levodopa (L-DOPA) is considered the standard of care for providing symptomatic relief in PD [1]. However, long-term L-DOPA treatment leads to the appearance of motor complications in the majority of responding patients and severely affects their quality of life [2]. After 9 years of L-DOPA treatment, ~90% of PD patients experience dyskinesia [3]. The dyskinesia that develops is often a combination of choreic and dystonic abnormal involuntary movements, collectively termed L-DOPA-induced dyskinesia (PD-LID).

Once PD-LID is established, increasing the L-DOPA dose typically worsens dyskinesia and this may prevent the use of L-DOPA at optimal doses required to control motor fluctuations. There are currently no licensed therapies for the treatment of PD-LID, although a number of clinical strategies are employed including adding dopamine agonists, monoamine oxidase inhibitors, adenosine (2A) receptor antagonists, catechol-O-methyl transferase inhibitors, and anticholinergic drugs as part of a L-DOPA-sparing strategy [4–7] and the use of amantadine [8]; see Tambasco et al. 2012 for a recent review [9].

The precise mechanisms of PD-LID are not completely understood, but excessive glutamatergic transmission within the basal-ganglia is thought to play a key role in the pathophysiology of PD and PD-LID [10, 11]. Therefore, therapeutic agents that regulate glutamate transmission are valid targets for drug development to alleviate motor symptoms associated with PD and PD-LID (Table 1). In this paper we will review attempts to develop therapeutic agents capable of normalising defective glutamatergic transmission via modulation of glutamate receptors.

## 2. Basal Ganglia Circuitry in Parkinson's Disease

A balance between inhibition and excitation of the major output nuclei of the basal ganglia is important for normal motor control. This is achieved via direct and indirect inhibitory projections (GABAergic) from the striatum (caudate nucleus/putamen) to the globus pallidus internal (GPi)/substantia nigra pars reticulate (SNr) and excitatory projections (glutamatergic) from the subthalamic nucleus (STN) to the substantia nigra pars compacta (SNc) and GPi/SNr. Appropriate dopaminergic input from the SNc to the striatum plays a key role in maintaining this balance [12]. In patients with PD, degeneration of dopamine nigral neurons within the SNc results in loss of dopaminergic modulation and increases the overall excitatory drive in the basal ganglia, disrupting voluntary motor control and causing the characteristic symptoms of PD [13]. The progressive depletion of the endogenous dopaminergic signalling combined with the compensatory exogenous supply of dopamine (DA) precursor L-DOPA induces profound changes in the neurotransmitter network of the basal ganglia [14]. An imbalance in the DA receptors, particularly between D1 and D2 receptor subtypes mainly expressed in the direct and indirect striatal output pathways, respectively, has been identified in dyskinetic nonhuman primates [15, 16]. In rodents, genetic ablation of the D1 receptor subtype but not the D2 subtype abolished the L-DOPA-induced dyskinesia. These results suggest a key role for the D1 receptor in the development of PD-LID [16]. However, a role for D2 receptors in the onset and expression of L-DOPA induced dyskinesias is also documented [17]. Additional changes in other dopamine receptor subtypes such as D3 and D5 have also been identified [18].

**Figure 1:**
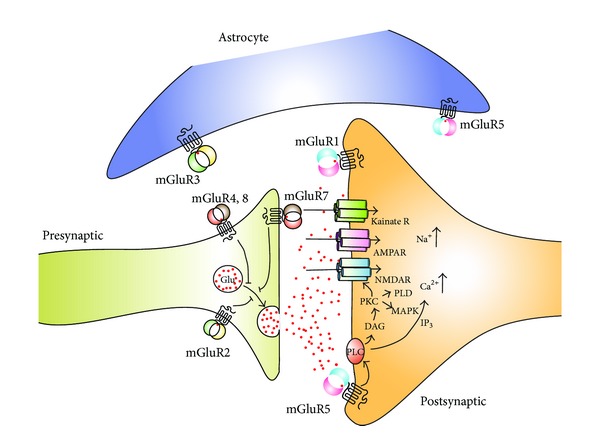
Schematic representation of ionotropic and metabotropic glutamate receptor subtypes, their intracellular function, and synaptic localization. AMPAR: *α*-amino-3-hydroxy-5-methyl-4-isoxazolepropionic acid receptor, DAG: diacylglycerol, iGluR: ionotropic glutamate receptor, IP_3_: inositol (1,4,5)-triphosphate, MAPK: mitogen-activated protein kinase, mGluR: metabotropic glutamate receptor, NMDAR: N-methyl-d-aspartate receptor, PKC: protein kinase C, PLC: phospholipase C, PLD: phospholipase D (reproduced with permission from Novartis Pharma AG. 2008 Novartis).

The cellular mechanisms by which the dopaminergic neurons are lost are not fully understood, although excessive glutamatergic transmission has been implicated [19, 20]. Glutamate is the main excitatory neurotransmitter in the CNS and normal brain function requires balanced glutamatergic neurotransmission. As a result of striatal dopaminergic denervation, the glutamatergic projections from the STN to the basal ganglia output nuclei become overactive with reduced regulation of glutamate receptors [19]. The resultant excessive excitation by glutamate through the basal ganglia circuitry can be toxic to any remaining dopaminergic neurons, leading to further loss of dopaminergic transmission and progression of PD symptoms [21]. Normalisation of motor function is initially seen with L-DOPA treatment. However, as the severity of PD increases, the substantial dopaminergic depletion leads to further adaptive changes in the basal ganglia pathways, including altered function of nondopaminergic basal ganglia neurotransmitters, such as glutamate, GABA, and serotonin [22]. Advanced nigral cell degeneration is considered responsible for the priming of the basal ganglia, as dyskinesia develops rapidly in monkeys with drug-induced nigral denervation following L-DOPA treatment [13], whereas L-DOPA treatment alone in animal models or healthy humans does not induce dyskinesia [23, 24]. Further evidence for a role for excessive glutamate transmission in PD and PD-LID comes from the clinical use of amantadine, a weak antagonist of NMDA glutamate receptors, in the treatment of PD-LID. To reduce the effects of the excessive glutamatergic transmission, several approaches aiming to modulate the glutamate receptors are available. These different approaches are dependent on the localisation and the function of each receptor subtype as illustrated on Figure 1 and will be described in the following sections.

## 3. Glutamate Receptors in Parkinson's Disease

Glutamate receptors modulate glutamatergic neurotransmission in the brain and play a role in memory, learning and motor control; glutamatergic dysfunction is implicated in a range of neurological disorders [25–27]. Two classes of glutamate receptor have been described: ionotropic glutamate receptors (iGlu receptors) [28], and metabotropic glutamate receptors (mGlu receptors) [26].

### 3.1. Ionotropic Glutamate Receptors. 

iGlu receptors are ligand-gated ion channels composed of four large subunits that form a central pore within the cell membrane. The iGlu receptors include the NMDA (N-methyl-d-aspartate), AMPA (*α*-amino-3-hydroxy-5-methyl-4-isoxazole propionic acid) and kainate ([2S,3S,4S]-3-[carboxymethyl]-4-prop-1-en-2-yl-pyrrolidine-2-carboxylic acid) receptors, all of which share a similar structure but differ in their amino acid sequences, subunit combination, and agonist sensitivity/selectivity. Within the CNS, iGlu receptors are responsible for fast excitatory transmission [28]. Their important role in mediating glutamatergic neurotransmission identifies the iGlu receptors as potential therapeutic targets for symptomatic management in PD and PD-LID.

#### 3.1.1. AMPA Receptors

Within the mammalian CNS, the majority of fast excitatory synaptic transmission is mediated by AMPA receptors [29]. AMPA receptor function is critical for synaptic plasticity. The potential for therapeutic AMPA modulation for PD is yet to be conclusively proven. Preclinical studies of AMPA receptor antagonists 6-nitro-sulfamoyl-benzo-quinoxaline-dione (NBQX) and GYKI-52466 failed to show antiparkinsonian activity when they were administered alone [30–34]. NBQX has shown some antiparkinsonian activity in another study where it reversed reserpine-induced muscle rigidity, but not akinesia in monoamine-depleted rats, and motor deficits in MPTP-lesioned Rhesus monkeys [35]. In another study, NBQX was combined with the competitive NMDA receptor antagonist 3-carboxy- piperazin-propyl phosphonic acid (CPP) and reversed the shortened duration of L-DOPA-induced motor responses [36]. Interestingly, NBQX [31, 32, 34] and CPP [30, 31] both potentiate the antiparkinsonian effects of coadministered dopaminergic agents. L-DOPA-sparing effects have also been shown in preclinical models with GYKI-52466 and GYKI-53405 [37, 38].

Increased AMPA receptor activity has been implicated in the development of LID [11, 39]. The competitive AMPA/kainate receptor antagonist LY293558 (tezampanel) reversed and prevented LID in parkinsonian rats [40] and the noncompetitive AMPA/kainate antagonist LY300164 (talampanel) decreased LID in MPTP-treated monkeys by up to 40% [41]. Another non-competitive AMPA receptor antagonist, perampanel, showed promising results in preclinical studies, reducing L-DOPA-induced motor defects 6-OHDA-primed rats [42] and dyskinesia in MPTP-treated monkeys [43]. Unfortunately, these results have not successfully translated to the clinical setting, and initial phase II and III trials of perampanel were terminated because of lack of efficacy [44–46].

#### 3.1.2. NMDA Receptors

NMDA receptors mediate glutamatergic excitation in the striatum and STN; NMDA-mediated signalling in the brain is thought to be involved in both neural plasticity and neurotoxicity [24]. Dysfunctions in NMDA receptor trafficking in striatal neurons result in the altered synaptic plasticity seen in animal models of PD and dyskinesia [47]. Several antagonists of NMDA receptors have shown therapeutic potential in animal models of PD: neuroprotective activity, by limiting the extent of nigrostriatal damage [48], or behavioural effects through the improvement of motor symptoms of PD [49, 50] and preventing or reducing LID [51–55]. Administration of competitive NMDA receptor antagonists such as CPP, CGP-43487, and APV, which target the glutamate binding site, or PAMQX, which binds to the glycine site in NMDA heterodimers, has also been shown to potentiate dopaminergic therapies in preclinical PD models [34, 56, 57]. There is also evidence of synergism between AMPA and NMDA antagonists in animal models of PD and LID [31, 58]. Further evidence of crosstalk between receptors involved in PD pathophysiology comes from studies showing interactions with 5-HT (2A) receptors [59, 60] and adenosine (2A) receptors in animal models [61], raising the possibility of adenosine (2A) or 5-HT (2A) receptor modulation as a novel therapeutic strategy for PD. NR2B-selective, non-competitive NMDA receptor antagonists, tnaxoprodil and ifenprodil, have shown therapeutic potential in animal models of PD-LID [50, 52, 62, 63].

Despite positive results in preclinical studies, clinical development of NMDA antagonists has been hampered by the side effects of these compounds, including psychosis, impaired learning, and disruption of motor function [64], which pose substantial problems with chronic use. This observed absence of a therapeutic window is likely due to the wide expression of NMDA receptors throughout the CNS and their key involvement in many physiological processes.

The greatest success with NMDA antagonists in PD and PD-LID has been seen with amantadine, a weak non-competitive NMDA receptor antagonist. Amantadine is approved for the treatment of PD and is widely used to treat PD-LID (off-label indication), due to its inclusion in international guidelines [65, 66]. Antidyskinetic activity with amantadine has been reported in both 6-OHDA rodent and MPTP primate models of LID [60, 67, 68]. Clinical benefits with amantadine in PD-LID have been seen in an increasing number of clinical trials [69–74] and the benefits appear to be long lasting [75, 76]. An extended-release formulation of amantadine, amantadine ER (ADS–5102), is currently in clinical trials for PD-LID. Other non-competitive NMDA receptor antagonists that have been investigated in the clinical setting include remacemide, which has failed to show benefit in the symptomatic management of PD and LIDs [77–80]. The non-competitive NMDA receptor antagonist, traxoprodil has shown clinical efficacy in two small studies [81, 82].

#### 3.1.3. Kainate Receptors

The existence of kainate receptors has been known for some time, yet little is understood about the contribution to the pathology of PD or the potential of kainate receptors as therapeutic targets in PD and PD-LID. This is primarily due to a lack of selective pharmacological agents to help elucidate the complex molecular mechanisms underlying these conditions.

### 3.2. Metabotropic Glutamate Receptors

The limitations of targeting iGlu receptors in PD combined with the high expression of mGlu receptors in the basal ganglia and their diverse modulatory roles has raised the interest in mGlu receptors as alternative targets for modulating glutamate hyperactivity in PD [12, 27]. mGlu receptors belong to the G-protein-coupled receptor family and are membrane-bound and activated by extracellular ligands. Unlike the fast excitatory glutamatergic transmission mediated by iGlu receptors, mGlu receptors have a more modulatory role, responsible for fine tuning glutamatergic transmission [26]. Eight mGlu receptor subtypes have been cloned and classified into three groups according to sequence similarity, signal transduction mechanism, and pharmacological properties [26]. Group I (mGlu1 and 5) receptors are coupled to activation of phospholipase C and mediate postsynaptic excitatory effects. They are mainly located postsynaptically and modulate glutamate transmission through negative and positive regulation of potassium and calcium ion channels, respectively. Group II (mGlu2 and 3) and Group III (mGlu4, 6, 7, and 8) receptors are negatively coupled to adenyl cyclase and inhibit cAMP formation. Both groups are generally located presynaptically and inhibit neurotransmitter release primarily through modulation of calcium and potassium ion channels. Of these 8 subtypes, mGlu5 and mGlu4 receptors are of particular interest as therapeutic targets in PD given their expression and distribution in the basal ganglia.

#### 3.2.1. Group I mGlu Receptors

**Figure 2:**
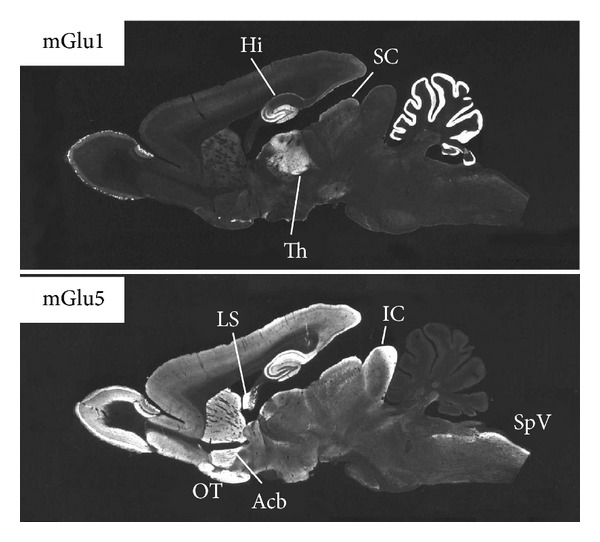
Immunolocalisation of mGlu 1 and 5 receptors within rat brain parasagittal sections. Acb, nucleus accumbens; Hi, hippocampus; IC, inferior colliculus; LS, lateral septal nucleus; OT, olfactory tubercle; SC, superior colliculus; SpV, spinal trigeminal nuclei; Th, thalamus [84].

Group I receptors mGlu1 and 5 share a high degree of sequence homology but their expression patterns within the brain differ considerably (Figure 2), suggesting that they may have distinct functional roles in brain physiology and pathophysiology.

The role of mGlu1 receptor in PD and PD-LID was examined using the selective antagonist, (3-ethyl-2-methyl-quinolin-6-yl)-(4-methoxy-cyclohexyl)-methanone methane sulfonate (EMQMCM), in 6-OHDA primed rats [83]. Treatment with EMQMCM induced some improvement of dyskinesia, altered downstream molecular signalling pathways, and attenuated L-DOPA-induced gene expression. However, these effects were achieved at a dose which blocked the antiakinetic (antiparkinsonian) action of L-DOPA. Together, these data do not support the use of mGlu1 receptor antagonists as a treatment for PD-LID.

The high expression of mGlu5 receptor in the caudate nuclei, putamen and basal ganglia [85, 86], and its postsynaptic localisation, make the mGlu5 receptor an attractive target to modulate the excessive glutamatergic neurotransmission induced by the loss of dopaminergic innervation. The first attempts to inhibit the mGlu5 receptor were made with competitive and nonselective antagonists. These early tool compounds had poor *in vivo *properties and brain penetration resulting in an inability to identify a role for the mGlu5 receptor. The identification and use of more specific tool compounds such as 2-methyl-6-(phenylethynyl)-pyridine (MPEP) enabled the hypothesis to be tested in various animal models of PD. The first confirmation of the potential to modulate excessive glutamatergic neurotransmission via inhibition of mGlu5 receptor came from the work of Spooren et al. [87]. They showed that MPEP could attenuate unilateral rotating behaviour in the rat 6-hydroxydopamine (6-OHDA) lesion model. Shortly after, Breysse et al. [88] provided further supportive data, by showing how chronic (but not acute) treatment with MPEP could improve akinesia in the 6-OHDA rat model. However, in this study no effects on haloperidol-induced catalepsy were observed following MPEP treatment [88]. In rats with nigrostriatal lesions, MPEP virtually abolished abnormal involuntary movements [89]. Using 3-[(2-methyl-1,3-thiazol-4-yl) ethynyl]pyridine (MTEP), which has superior specificity and bioavailability to MPEP, a reduction of haloperidol-induced catalepsy and muscle rigidity in rats was seen [90].

More recently, mGlu5 receptor expression was shown to be enhanced within the posterior putamen and globus pallidus of parkinsonian monkeys experiencing dyskinesia following chronic L-DOPA treatment [91] and in human postmortem brains of parkinsonian patients with dyskinesia and wearing off [92]. In addition, animal models of PD-LID show upregulation of mGlu5 receptor genes, reflecting long-term changes associated with the development of dyskinesia [93]. There is also evidence of crosstalk between mGlu5 receptors and NMDA receptors in the striatum and subthalamic nucleus [94, 95], and it is possible that therapeutic modulation of mGlu5 receptors may have beneficial effects on NMDA receptor signalling in PD. Additionally, mGlu5 receptors and adenosine A (2A) receptors are coexpressed in D2 striatal neurons and interact to regulate the downstream effects of mGlu5 receptor activity [96–99].

The first evidence for the therapeutic potential of mGlu5 antagonists in PD-LID was presented by Hill et al. [100] using the noncompetitive antagonist, SIB-1893, to ease LIDs in MPTP-lesioned monkeys. Interestingly, SIB-1893 was identified initially as a relatively weak mGlu5 receptor antagonist (IC_50_ = 2.3 *μ*M) [101], whereas further characterisation revealed SIB-1893 to also be a positive allosteric modulator (PAM) of the mGlu4 receptor [102].

The use of MTEP confirmed that antagonism of mGlu5 receptor attenuates LID in 6-OHDA lesioned rats [103, 104] and MPTP-lesioned monkeys [105]. In MPTP-lesioned monkeys, MPEP showed antiparkinsonian effects and reduced the development of LID [106]. Similar results were seen with both MPEP and MTEP in MPTP-lesioned monkeys treated with L-DOPA [105]. Other selective mGlu5 receptor antagonists that have shown preclinical anti-dyskinetic effects include 6,6-dimethyl-2-phenylethynyl-7,8-dihydro-6H-quinolin-5-one (MRZ-8676) [100], dipraglurant (ADX48621; Addex Press Release), and mavoglurant (AFQ056), which reduced dyskinesia in L-DOPA-treated MPTP-lesioned monkeys [100]. In addition, mavoglurant did not adversely affect the response to L-DOPA in MPTP-lesioned monkeys, but did potentiate the effects of low doses of L-DOPA [107].

In contrast to other therapeutic approaches in PD, the wealth of preclinical data supporting the potential of mGlu5 receptor antagonists in treating PD and LID has been confirmed clinically by two drug candidates, mavoglurant [108, 109] and dipraglurant [110]. Both have shown significant anti-dyskinetic activity in patients with moderate-to-severe PD-LID. Follow-up trials with both mavoglurant and dipraglurant are ongoing.

#### 3.2.2. Group III mGlu Receptors

Among the Group III mGlu receptors, mGlu4, 7, and 8 are expressed at multiple synapses throughout the basal ganglia and mainly localised presynaptically [12, 85]. Their activation inhibits neurotransmitter release, a mechanism implicated in the pathophysiology of PD [111–113]. Preclinical studies with selective Group III mGlu receptor competitive agonists reversed akinesia and haloperidol-induced catalepsy in rodent models of PD [114–116].

Similarly, PAMs targeting mGlu4 receptor have shown some antiparkinsonian activity in animal models of PD. For example, N-phenyl-7-(hydroxyimino) cyclopropa[b]-chromen-1a-carboxamide (PHCCC) reversed risperidine-induced akinesia in rats [117] and reduced striatal dopamine neuron degeneration in MPTP-treated mice [118]. However, PHCCC has low potency and poor aqueous solubility and demonstrates antagonism at mGlu1 receptors at a similar potency to that at mGlu4 receptors [117]. Therefore, agents exhibiting greater potency and selectivity for mGlu4 receptors have been sought to clarify the therapeutic potential of targeting this receptor subtype in PD. One such agent, VU0155041, has shown antiparkinson activity in haloperidol-induced catalepsy and 6-OHDA lesioned rats [119, 120]. VU0155041 also demonstrated synergy when coadministered with the adenosine (2A) receptor agonist preladenant, as well as L-DOPA, suggesting a potential L-DOPA-sparing mechanism [120]. Similarly,   5-methyl-N-(4-methylpyrimidin-2-yl)-4-(1H-pyrazol-4-yl) thiazol-2-amine (ADX88178), a PAM with high bioavailability and specificity for mGlu4 receptor, has antiparkinsonian activity including potentiation of L-DOPA effects, without increasing LID [121].

Activation of the mGlu7 receptor using the PAM, N,N′-dibenzhydrylethane-1,2-diamine dihydrochloride (AMN082), has also shown antiparkinsonian and antidyskinetic activity in rodent models of PD [12, 122]. Currently, there are no agonists or PAMs, with appropriate oral bioavailability and brain permeability available for targeting the mGlu8 receptor. However, intracerebroventricular injection of the mGlu8 receptor agonist, (S)-3,4-dicarboxyphenylglycine (DCPG), reportedly reversed parkinsonian symptoms in a rat model for PD [123].

#### 3.2.3. Group II mGlu Receptors

Activation of Group II mGlu2 and 3 receptors using competitive agonists has been extensively characterised in animal models. Based on their preclinical profile, these group II agonists have been evaluated in the clinic for anxiety [124] and schizophrenia [125]. In contrast, preclinical data supporting a beneficial effect in PD are limited. Both receptors have been located in key areas of the basal ganglia associated with PD pathophysiology such as the SNr, and their activation is known to inhibit synaptic excitation [85, 126, 127]. However, ligand binding autoradiography in postmortem brain tissue, from MPTP-treated monkeys and patients with PD, suggests that there are no clear changes in the expression of mGlu2 and 3 receptors associated with PD-LID [128, 129]. Systemic administration of the competitive mGlu2/3 receptor agonist LY379268 failed to provide any functional benefit in the 6-OHDA lesioned rat model [130]. Johnson et al. suggested that [131] the lack of subtype specificity and limited brain penetrability of LY379268 were responsible for the absence of efficacy seen following systemic administration [122]. PAMs selective for mGlu2 receptor have an improved pharmacokinetic profile and good brain penetration [123]. *In vivo*, mGlu2 receptor PAMs have shown that they are valuable alternative to the competitive agonists in a rodent model for panic-like behaviour [132]. Future investigations of PAMs targeting mGlu2 receptors using appropriate animal models will be key to evaluating the potential of this mechanism of action in PD.

## 4. Conclusions

Nigrostriatal denervation in PD leads to increased glutamatergic transmission in the basal ganglia, which leads to further loss of dopaminergic neurons, progression of PD, and the appearance of PD-LID. Pharmacological modulation of glutamatergic transmission is a key focus for research into novel nondopaminergic agents for PD. Antagonists of iGlu receptors have shown antiparkinsonian and antidyskinetic effects in preclinical studies, but the emergence of adverse effects has limited the clinical value of these agents. Amantadine, a weak NMDA antagonist, is the exception, showing significant anti-dyskinetic effects in the clinical setting. Modulators of mGlu receptors hold greater promise in PD due to their location in the basal ganglia and the development of a number of agents with high potency and selectivity for different mGlu receptor subtypes. In particular, preclinical studies with mGlu5 receptor subtype selective antagonists and PAMs of mGlu4 receptor have shown good efficacy in models of both PD and PD-LID, and there is now growing clinical evidence for mGlu5 receptor antagonism as a valid therapeutic target for PD-LID. Research into glutamate receptor signalling in the basal ganglia is now revealing a hugely complex network involving cross-talk between different glutamate receptors, dopamine, and adenosine receptors. As we understand more about the importance of these interactions, we may be able to develop compounds that can fine tune dopaminergic and non-dopaminergic transmission, leading to better treatments for PD and PD-LID.

## Figures and Tables

**Table 1:** Glutamatergic agents cited within the text with their pharmacological profile, main target/s, and mode of action.

Target	Agent	Pharmacological action
AMPA receptor	GYKI-52466	Noncompetitive antagonist
	GYKI-53405	Noncompetitive antagonist
	NBQX	Competitive antagonist
	Perampanel	Noncompetitive antagonist

AMPA/kainate receptor	Tezampanel (LY293558)	Competitive antagonist
	Talampanel (LY300164)	Noncompetitive antagonist

AMPA/NMDA receptor	CPP	Competitive antagonist

NMDA receptor	Amantadine	Noncompetitive antagonist
	APV	Competitive antagonist
	CGP-43487	Competitive antagonist
	Ifenprodil	Noncompetitive antagonist
	PAMQX	Competitive antagonist
	Remacemide	Noncompetitive antagonist
	Traxoprodil	Noncompetitive antagonist

mGlu1 receptor	EMQMCM	Noncompetitive antagonist

mGlu2 receptor	LY379268	Competitive agonist

mGlu4 receptor	PHCCC	PAM
	ADX88178	PAM
	VU0155041	PAM

mGlu4/mGlu5 receptor	SIB-1893	PAM/noncompetitive antagonist

mGlu5 receptor	Dipraglurant (ADX48621)	Noncompetitive antagonist
	Mavoglurant (AFQ056)	Noncompetitive antagonist
	MPEP	Noncompetitive antagonist
	MRZ-8676	Noncompetitive antagonist
	MTEP	Noncompetitive antagonist

mGlu7 receptor	AMN082	PAM

mGlu8 receptor	DCPG	Competitive agonist
